# Advances in Smart Environment Monitoring Systems Using IoT and Sensors

**DOI:** 10.3390/s20113113

**Published:** 2020-05-31

**Authors:** Silvia Liberata Ullo, G. R. Sinha

**Affiliations:** 1 Engineering Department, Università degli Studi del Sannio, 82100 Benevento, Italy; 2 Myanmar Institute of Information Technology (MIIT), 05053 Mandalay, Myanmar

**Keywords:** environment, pollution, internet of things (IoT), sensors, smart environment monitoring (SEM), smart sensor, wireless sensor networks (WSNs)

## Abstract

Air quality, water pollution, and radiation pollution are major factors that pose genuine challenges in the environment. Suitable monitoring is necessary so that the world can achieve sustainable growth, by maintaining a healthy society. In recent years, the environment monitoring has turned into a smart environment monitoring (SEM) system, with the advances in the internet of things (IoT) and the development of modern sensors. Under this scenario, the present manuscript aims to accomplish a critical review of noteworthy contributions and research studies on SEM, that involve monitoring of air quality, water quality, radiation pollution, and agriculture systems. The review is divided on the basis of the purposes where SEM methods are applied, and then each purpose is further analyzed in terms of the sensors used, machine learning techniques involved, and classification methods used. The detailed analysis follows the extensive review which has suggested major recommendations and impacts of SEM research on the basis of discussion results and research trends analyzed. The authors have critically studied how the advances in sensor technology, IoT and machine learning methods make environment monitoring a truly smart monitoring system. Finally, the framework of robust methods of machine learning; denoising methods and development of suitable standards for wireless sensor networks (WSNs), has been suggested.

## 1. Introduction and Background

Sustainable growth of the whole world depends on several factors such as economy, quality education, agriculture, industries and many others, but environment is one of the factors that plays the most important role. Health and hygiene are key components of the sustainability of mankind and progress of any country, which comes from a clean, pollution free and hazardous free environment. Thus, its monitoring becomes essential so as to ensure that the citizens of any nation can lead a healthy life. Environment monitoring (EM) consists of proper planning and management of disasters, controlling different pollutions and effectively addressing the challenges that arise due to unhealthy external conditions. EM deals with water pollution, air pollution, hazardous radiation, weather changes, earthquake events, etc. The sources of pollution are contributed by several factors, some of which are man-made and others due to natural causes, and the role of EM is precisely to address the challenges so that the environment is protected for a healthy society and world [1]. With the more recent advances in science and technology, especially artificial intelligence (AI) and machine learning, EM has become a smart environment monitoring (SEM) system, because the technology has enabled EM methods to monitor the factors impacting the environment more precisely, with an optimal control of pollution and other undesirable effects. The design of smart cities is taking the place of old and traditional methods to create and plan urban environments. Smart cities are planned using wireless networks that assist monitoring of vehicular pollution level in the city [2]. Wireless networks or wireless sensor networks (WSNs) comprise modern sensors which operate on AI based monitoring and controlling methods. Internet of things (IoT) devices are employed in WSNs for effective waste management, vehicle marking, temperature control, and pollution control. Therefore, modern methods of environment monitoring are known as SEM systems, due to use of IoT, AI and wireless sensors [3]. Assessment of burned areas using multispectral data captured through satellite imaging and remote sensing [4], mobile health monitoring systems and IoT based environment systems [5], smart marine environment systems using multimodal sensing networks [6], and many other SME methods are reported in current literature. When wireless devices are used over a WSN, then certain standards and protocols are important for effective implementation of SEM systems and thus studies are also reported on developing protocols and standards for IoT based SEM systems [7].

The whole world is working in a comprehensive manner to protect the environment for sustainable agriculture, growth and a healthy society and therefore the main aim of SEM is to address the challenges due to undesirable effects in the environment through smart monitoring so that all key indicators of growth, including the health of society, are well regulated. The environment monitoring methods are implemented for various applications, aiming to serve certain purposes, which may include weather forecasting [8,9], air pollution control [10,11,12], water quality control and monitoring [1,13,14], and crop damage assessment [14,15], for instance. The objective is to facilitate favorable environment conditions either for agriculture or human beings, or any inhabitants on the earth. The technologies such as IoT and wireless networks have made the monitoring of environment simple and AI controlled. The SEM systems are reported in the literature using different types of smart sensors [8,16,17,18,19], wireless sensor networks (WSNs) [11,14,18,20,21,22], and IoT devices [1,3,5,8,10,18,23,24]; these devices, communicating through the networks, have helped the environment monitoring as a smart monitoring system, able to address the challenges in variable conditions.

**Figure 1:**
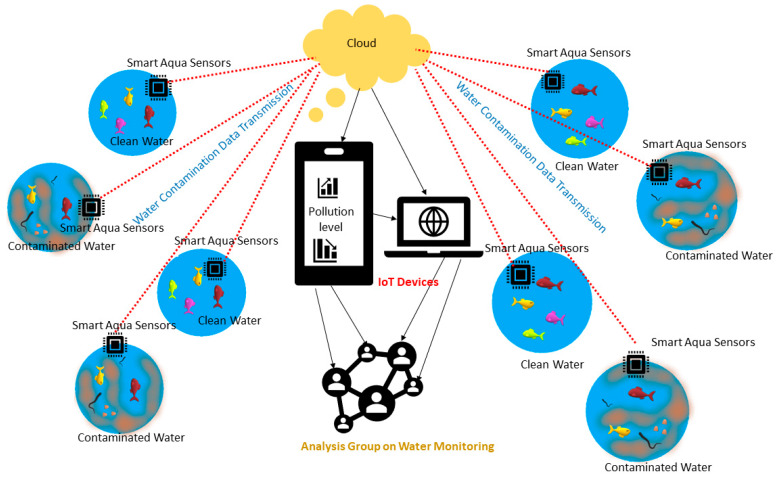
Smart environment monitoring (SEM) system highlighting water contamination and its monitoring using the cloud connecting internet of things (IoTs) and sensors.

IoT, WSNs and suitable sensors are the backbone of the SEM systems. The WSNs provide the connectivity of the data, captured by employing sensors and IoT devices, used to record, monitor and control various environmental conditions, such as water quality, temperature, air quality, etc. A smart environment system can be easily understood with the help of an example of a cloud based SEM system, as shown in Figure 1. The example shown in this figure depicts monitoring of water contamination and its control, by using a cloud based system that connects IoT devices and various suitable sensors. The system can monitor, with the help of IoT devices, if the water is contaminated or clean since all IoT devices have embedded the capability of AI and machine learning. The organization, which is involved in monitoring the water quality of various water sources, has access to the cloud through the data collected from various sensors, for example an aqua sensor, and is subjected to IoT based analysis where the quality check is done.

**Figure 2:**
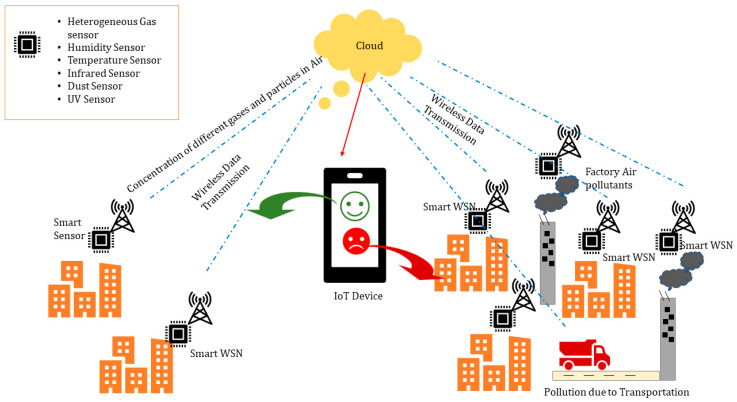
SEM system addressing various issues in the environment using wireless sensor networks (WSNs) and IoT devices.

One more example of a SEM system, highlighting a general purpose system with extended scope, is shown in Figure 2., which shows how the system is addressing various issues related to environment monitoring, such as humidity, temperature, radiation, dust, UV signal etc. The backbone of the system is a WSN that is establishing the actual interface between IoT devices and data captured through various types of smart sensors. This is a perfect example of a “smart city” [11,25,26], using a SEM system that ensures healthy environment for its citizen.

**Figure 3:**
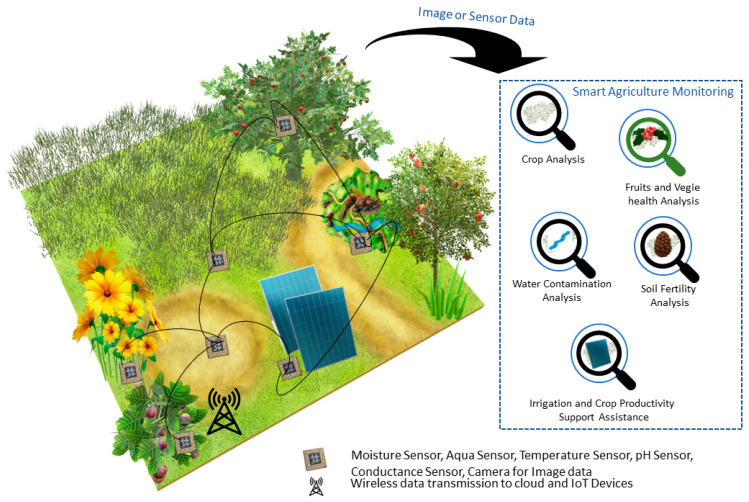
Smart agriculture monitoring system using IoT devices and sensors.

By focusing on agriculture, as a relevant issue for the growth of any nation, it is easy to underline how SEM can play a significant role by providing a “smart or green agriculture” [14,20,27,28], that can deal with major challenges and factors involved in sustainable growth and enhancing productivity within the agriculture sector. One such smart agriculture scenario can be seen in Figure 3, where a SEM system is actually a smart agriculture monitoring system. In this case, the health of soil, moisture analysis, water contamination level, water quantity level and several other factors are very important in obtaining sustainable productivity in the agriculture sector. We can see in Figure 3 that the smart agriculture monitoring system includes all such factors, controlled and monitored with the help of IoT devices, suitable sensors capturing the agricultural data, then transmitted to the cloud through a WSN. 

We attempted to study the existing contributions by a critical survey on SEM methods; the literature suggests that the extensive reviews on SEM methods which have discussed significant findings are not found. We could not find much literature that reviews or surveys SEM techniques. A survey on smart agriculture systems [29], smart home technologies [30], smart health monitoring systems [31], environment monitoring [32], an IoT based ecological system [33], IoT for marine environment monitoring [34], and a survey on pollution monitoring system [35], are a few of the survey and review related articles highlighting different aspects of SEM. The environment is contaminated due to several factors, but water pollution, air pollution, radiation and sound pollution are mainly involved in most of the existing research. This motivates us to bring out an extensive review on SEM that covers all important factors affecting the health of the environment and predominant methods used to mitigate the challenges due to these factors, such as IoT and sensor technologies. 

We have briefly discussed in this section the main issues related to environment monitoring, SEM, the role of IoT, AI and WSNs in implementing SEM. The next part of the paper is organized as follows: Section 2 discusses related research and study; Section 3 presents comparative analysis of advances in SEM systems; Section 4 highlights the significance of the study and recommendations). 

## 2. Related Research and Study

The current research suggests that environment monitoring systems are implemented smartly as SEM for various purposes and using different methods. A huge number of contributions on SEM, both based on purposes and types of methods, have been studied and therefore the related research has been discussed in three main subsections, namely the study based on smart agriculture monitoring systems (SAMs), smart water pollution monitoring systems (SWPMs), and smart air quality monitoring systems (SAQMs). In this manuscript the authors have attempted to critically report the major findings and limitations of the current research on SEM. Soil monitoring (SM) [14,15,36], ocean environment monitoring (OEM), marine environment monitoring (MEM), air quality monitoring (AQM) [10,11,37,38], water quality monitoring (WQM) [14,39], and radiation monitoring (RM) [1,36] have been covered, by offering a wide analysis of different application fields of SEM.

While studying the existing literature on SEM methods, especially on advancements in IoT and sensor technologies for SEM systems, we found that an extensive review on this topic has not been much reported. We found some interesting literature on specific areas of research addressing some challenges of environmental factors such as water pollution, air quality, radiation, and smart agriculture. We aimed at bringing out major advances in IoT and sensor technologies used for addressing the challenges in SEM and thus we included some significant research studies and contributions of various sources highlighting specific classic work on SEM methods. The current study on advances in IoT and sensor technologies used for SEM provides insight to the scientists, policymakers, and researchers in developing a framework of appropriate methods for monitoring the environment that faces challenges mainly due to poor air quality, water pollution and radiation. These factors also affect agriculture which is backbone of any developed and developing economy and thus smart agriculture monitoring (SAM) has also been studied in this section. 

Table 1 shows major research studies and contributions on the above SM, OEM, MEM, AQM, WQM and RM areas of interest. Soil monitoring methods were reported to have been affected by greenhouse effects. Ocean and marine SEM systems have been implemented using sensors, WSN and IoT and these methods have mainly suffered with cost, coverage and installation issues [40,41,42]. Air pollution control and AQM [1,10,11,16,43,44,45] have been suggested using a mobile sensor network, wireless sensors and IoT devices that operate on AI and machine learning. In a similar manner, we can see in Table 1 that the different types of SEM systems are designed and implemented for various purposes and there is no robust method that can address any of the challenges of environment. 

### 2.1. Study based on Smart Agriculture (SAM)

This section presents studies and research on smart agricultural monitoring (SAM) systems covering the measures for crop monitoring, pest control, fertilizer control etc. The research study summary for a few important works can be seen in Table 2. Plant growth monitoring [54] was implemented and named as “gCrop”, using IoT, machine learning and WSN. The work uses a regression model of the 3^rd^ degree and provides a prediction accuracy of 98% but the computational complexity was high. The analysis of crop quality [14,46] assessment was made using SAR data for monitoring the quality of paddy rice. Support vector machines (SVMs) with back-scattering features were used in this assessment of the rice quality, with a limited sample size. Leaf area and dimension also play an important role in the assessment of various types of crops, as means to determine if the growth is satisfactory or not. One such work was reported in [55], that was used to measure the leaf area index using SVM as the machine learning technique, with a Gaussian process model [56] and the accuracy of measurement found as 89% with a limited sample size also in this case. An expert system using AI has been implemented in [57] using the Naive Bayes [58] method and machine learning which operates on sensor data captured in agriculture. This work was useful in monitoring the quality of fertilizer, pesticides and the amount of water to be irrigated in the crops. Some other works studied crop quality assessment [21,59,60] and [61] used for monitoring of the soil health, suitable for soya bean crop on the basis of phenological data and unmanned aerial vehicle (UAV) real-time images. There are a few other important studies on various application of SEM systems for different applications, such as smart farming [62], pest monitoring [63], and crop area monitoring [61].

The environment conditions affect the health of crops and consequently the agriculture growth. Therefore, we aimed at studying the status of research on SEM using IoT, sensors and AI techniques. The factors involved in agriculture such as soil condition, moisture condition, water pollution, air quality, temperature etc. have been taken into consideration while reviewing the advances in SEM methods. The focus is given to studies on water pollution monitoring and air quality monitoring methods also, which are discussed in next sub-sections.

### 2.2. Study based on Smart Water Pollution Monitoring (SWPM) Systems

Different literature has been studied on smart water pollution monitoring (SWPM) methods and systems using machine learning methods, IoT and wireless sensors. Table 3 depicts a few major contributions in the area of SWPM. Remotely sensed images were analyzed and machine learning was applied for prediction of the pollution level in the lagoon water, useful for agriculture [64]. This work used ordinary neural network based machine learning and the prediction results were not very satisfactory. Classification of water contamination [65] has been studied and water was classified as clean or polluted water, using machine learning methods and IoT devices. The paper presented a realtime contamination monitoring system, though the data captured were in a limited area only. The assessment of various pollutants mixed in water has been implemented in [66] and the pollutants were classified using a DSA-ELM model [66] with the evaluation of the model itself. AI and neural network based prediction of water quality parameters was studied in [67], and alkalinity, chloride and sulphate contents were estimated. The work mainly focused on prediction of water quality parameters and values of sulphate or chloride present in the water. Big data analysis and issues in classification of water contamination were discussed for classification of the contamination using SVM in [68]. Quality assessment of drinking water and its classification into drinkable and non-drinkable water were presented in [69,70], as a real time monitoring system and AI-SVM based classification technique respectively. A video based surveillance of water quality and pollutants, was studied in [71], and the surveillance helped to stop the man–man sources of pollutants. The work employed IoT tools for video-surveillance and machine learning for classification of water as polluted and clean water. One more work on a drinking water prediction model was suggested in [61], and a feature based model helped in analysis of drinking water to further predict its quality before usage. In another work, chlorophyll-A concentration in lake water was assessed using different machine learning models [72], and the work was recommended as method for a realtime lake water cleaning management system.

### 2.3. Study based on Smart Air Quality Monitoring (SAQM)

Research on SAQM methods and systems have also been studied, and Table 4 presents a summary of different SAQM approaches used in recent literature on air quality monitoring systems. Air quality characterization [58] has been implemented using heterogenous sensors and machine learning methods. The monitoring as well as characterization of water quality was achieved but interoperability issues were reported in this work due to use of heterogenous sensors. Air quality evaluation using fixed as well as mobile nodes of sensors [75] was implemented, capable to check the air quality in stationary as well as mobile ways. In this latter case, the compatible sensors were deployed as mobile nodes which can work satisfactorily in a moving environment. Data captured through smart sensor nodes were processed and analyzed with the help of machine learning techniques. Another air quality control process was studied using IoT and machine learning techniques in [76], with a focus on assessment of air pollution, deploying gas sensors which help in capturing air particles and analyzing the pollutants mixed in the air. Sensor networks have been established in moving vehicles for monitoring air quality with the help of machine learning; in [77], mobile sensor nodes and WSN were deployed. Infrared sensors were deployed to evaluate the air quality, especially analyzing volatile organic compounds (VOCs) in [25], with the help of machine learning methods. The elements of VOCs were detected and analyzed using spectroscopic observations. There are a few components present in the air that help assessing the quality of the air; one such component, called PM2.5, was predicted in [78], using extreme machine learning techniques tested upon spatio-temporal data collected in a certain duration of time over a range of distances covered by the sensors. Different forecasting models were suggested in [44] for quality evaluation of urban air and the components like O3, SO2 and NO2 were determined and a comparison was made for the models used in the work. RFID and a gas sensor based air quality control mechanism were implemented in [79], to determine the level of pollution in the air by predicting the pollution value; IoT was employed to analyze sensory data captured through gas sensors. RFID was primarily used in this work for detection of pollutants and communicating to WSNs with the help of IoT devices connected across a WSN architecture. An SAQM system has been studied in [80], using a LoRaWAN (long range WAN) [80,81,82], and this work has been very useful for detecting temperature, dust, humidity and carbon dioxide components in the air. An intelligent air quality system was presented for detection of CO2, NOx, temperature and humidity in [83] using AI and machine learning techniques for developing expert systems for air quality assessment. Furthermore, PM10, PM2.5, SO2, oxides of nitrogen (NOx), O3, lead, CO and benzene components were detected, on the basis of machine learning methods trained by spatio-temporal data, in [84]. This was extended using deep learning for detection and detailed analysis of O3 components only. Another work employing heterogenous sensors was studied in [85]. SVM was used for analyzing the sensor data, captured through heterogenous sensors, and air quality was estimated.

## 3. Discussion, Analysis and Recommendation

This section presents analysis, discussion and a few significant recommendations on the basis of extensive literature review on various SEMs. The SEM systems were studied, covering air quality assessment, water pollution monitoring and agriculture monitoring system, in addition to the sub-subsidiary applications of these three major studies. The recent research contributions were the main focus of the study though a few important research studies, conducted and investigated in last two decades, were also included. The contributions were reported on various SEM methods used for several purposes, mainly air quality assessment [1,5,11,12,47,58,76,85,89,90]; water pollution monitoring methods [1,13,14,39,64,66,71,72,73,91,92,93,94,95,96,97]; radiation monitoring methods [1,36]; and smart agriculture monitoring systems [1,14,28,54,60,62,63,98,99,100,101,102].

The extensive study on SEM methods brings out the following major observations for the discussion:

- The research on SEM includes various purposes, mainly on SAM, SWPM and SAQM. The study of water pollution, air quality, soil moisture and humidity can help in modeling and design of healthy environment systems that would also help smart agriculture for sustainable growth of the economy.
- The methods under each of the purposes are divided in terms of sensory data used, machine learning methods used, IoT devices used, and types of sensors involved. The current study made by us mainly focused on impact of existing research on water quality monitoring, air quality assessment, applications of SEM and smart agriculture systems.
- In most of the SEM methods, especially SAM and SWPM, CNN based deep learning methods are used by the researchers and other deep learning models are not very frequently used.
- The sensory data vary in most of the applications of SEM and there is no robust data over which a maximum number of methods are operating. The data type and regions of interest are not the same for various research work.
- The methods have been used for either classification or prediction; for example, water is classified as polluted or clean water; similarly, the water and air quality can be predicted (e.g., level of degradation).

The studies reported for all purposes of SEM systems do not have any common challenges and vary from application to application, but the major challenges observed are as follows:

- Wherever heterogeneous sensors are used, there is problem of interoperability in the analysis of the data captured through different types of sensors.
- Sample size is limited in many of the contributions.
- Noisy data poses a challenge in analysis. Noise is present in the data captured through sensors used for various purposes. The noise may be contributed by several internal and external factors.
- The machine learning methods which have been employed for training the data and for classification are mostly traditional methods of machine learning, such as SVM, neural network, etc.
- Fuzzy based methods and deep learning approaches are used in a few research studies and implementations, but the research suffers with either big data issues or huge computational complexity.
- There is no robust approach of machine learning reported, that can be employed in addressing the challenges of the environment irrespective of the purpose of the monitoring and control, types of data, and types of sensors used.

Research trends were also analyzed to assess the quantum of research carried out in the area of SEM [14,21,31,103,104,105,106,107,108,109,110,111,112,113] and Table 5 shows a summary of quantity of research in this case. The study of trends was made by using a publication search in the Science Direct databases in year-wise manner. In this analysis, the duration has been chosen from year 1995 to year 2020. It can be clearly seen in Table 5 that the quantum of research on SEM has been increasing with the time in both the case, namely the research employing IoT and WSN, as well as the research using IoT and machine learning. An interesting fact is an outcome of the table: the research using modern machine learning methods is still lagging behind those which do not use any machine learning. However, if IoT devices are used, and deployed in a WSN, then the role of AI cannot be overlooked.

**Figure 4:**
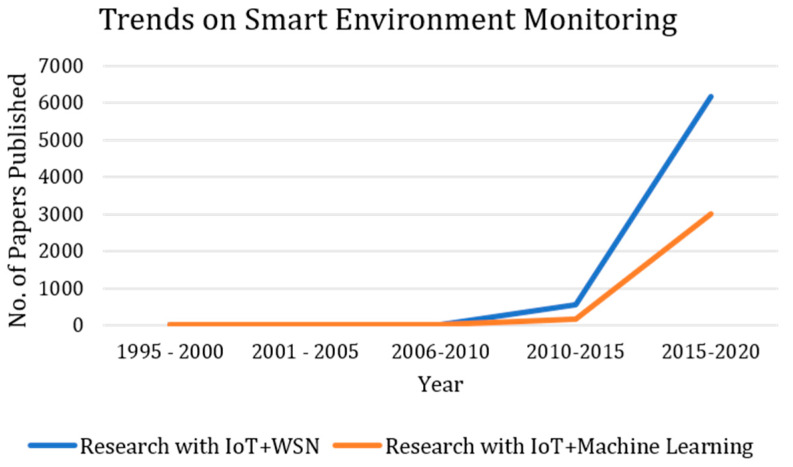
Trends of SEM methods.

The analysis of research trends are shown in Figure 4, highlighting the research trends in two main categories, namely SEM using IoT and WSN, and IoT and machine learning, respectively. The trends suggest that the SEM has yet to be implemented and studied widely on machine learning based training and subsequent classification or prediction. The research is reported to have increased every year but more impact of IoT and WSN can be seen in Figure 4.

The above discussion and analysis helps us recommending the following for better, robust and smarter environment monitoring systems:

- A framework of machine learning methods needs to be developed.
- A robust set of classification, prediction and forecasting models has to be designed that can operate on any data, irrespective of the purpose of using the SEM.
- Suitable denoising methods are required to be implemented as pre-processing to the SEM major stages, since most of the research has failed using de-noising the data and its appropriate pre-processing.
- Data deduplication approaches and other methods are needed to deal with big data issues involved in a few significant studies.
- SEM aims at sustainable development of any nation and the smart agriculture and smart environment play a most important role in achieving the sustainable goals, but in rural areas, in most of the developing and underdeveloped nations, the necessary infrastructure for setting up IoT, WSN and other sensors is still a challenging task. This requires governmental level involvement both at local as well as global perspectives.
- Interoperability issues in implementing various types of sensors, can be addressed by developing suitable standards and protocols that can make the data compatible for all acquisition and analysis systems.

An attempt was made to include major observations of a few significant review articles on SEM but it was very difficult to report any such extensive review on the SEM in particular. This motivated us to study the most important contributions on research addressing environmental challenges due to main factors. This review helped us to reach to some conclusions and make recommendations for designing a robust SEM systems that can handle all possible challenges using a framework of AI and sensor technologies.

## 4. Conclusions and Future Scope of Work

This paper has presented an extensive and critical review of research studies on various environment monitoring systems used for different purposes. The analysis and discussion of the review suggested major recommendations. The need of extensive research on deep learning, handling big data and noisy data issues, and a framework of robust classification approaches has been realized. We have focused mainly on water quality, and air quality monitoring as smart agriculture systems that can deal with environmental challenges. The major challenges in implementation of smart sensors, AI and WSN need to be addressed for sustainable growth through SEM. The participation of environmental organizations, regulator bodies and general awareness would strengthen SEM efforts. The poor quality of sensory data can be preprocessed using appropriate filters and signal processing methods to make the data more suitable for all subsequent tasks associated in SEM. The future scope of the work aims at studying other factors of environment such as sound pollution and disasters etc.

## Figures and Tables

**Table 1:** Research studies based on purpose and applications of environment monitoring.

Research	Purpose	Findings and Challenges	Method/Device Used
**OEM** [40]	Oceanic environment monitoring	Light weight; costly and invasive sensory networks	Wireless Sensors
**IOT Based SM** [46]	Soil monitoring for farming	Efficient vegetable crop monitoring; Greenhouse gases pose challenges on health of vegetables like tomato	Wireless sensors
**IoT Protocols for MEM** [42]	Marine environment acoustic monitoring	Lower latency; low power consumption; installation and coverage issues	WSN and IoT
**IoT for air pollution** [47]	Air pollution monitoring system	Mobile kit “IoT-Mobair” for prediction; inferior precision; low sensitivity; computationally complex	Gas sensor and IoT
[5]	Air quality monitoring	Scalable and high-density air quality monitoring with interconnection of heterogeneous sensors; computational complexity due to huge data captured and processed	Mobile sensor network and WSN
**IoT based SEM** [7]	Environmental monitoring	W3C standard for interoperability; interoperability issues of heterogeneous sensors	Heterogeneous sensors
**Air quality** [12]	Air quality monitoring	Large area monitoring; noisy data; accuracy and cost issues	Geomatics sensors and IoT
**Pollution monitoring** [16]	Air pollution monitoring System	Real time monitoring; accuracy issues	Sensors with MQ3 Model, Raspberry Pi and IoT
**Sensor based AQM** [37]	Air pollution monitoring system	Efficient for low coverage area; low cost; easy to install; less number of pollutants are covered	Gas sensor and LASER sensor
**SEM** [48]	Dust and humidity monitoring	Wide coverage and efficiency; low cost and small size	IoT
**Radiation** [36]	Radiation monitoring	High cost and low stability against temperature variation	HPXe chamber
**Aqua farming and energy conservation** [49]	Aqua Farming	Water quality and quantity control; higher carbon emission and energy requirement	Odor, pH, conductance and temperature sensor
**Multi-agent supervising system** [50]	e-health monitoring system due to temperature and radiation changes around the surroundings	Detection of emergency situations	Supervising system and AI
**SEM in winter season** [51]	Effect of surroundings during winter season only	Effect of batteries and other radiation	Wireless sensor network
**LoRa technology for climate monitoring** [32]	Climate and ecology monitoring	Study of emissions in the environment	LoRa technology and sensor network
**Smart city and SEM** [52]	Monitoring of data center radiation	Temperature, humidity and energy consumption in data centers monitored for smart city and SEM	IoT
**ZigBee based environment monitoring** [53]	Smart industry environment	To study hazardous effects in industries	ZigBee and WSN LoRa: Long Range

**Table 2:** Research on IoT based SEM systems.

Purpose/ Area of Study	Device/Method Used	Models
**Plant growth** [54]	IoT, WSN, Machine learning based “gCrop” (green-crop)	Regression model of 3rd degree of polynomial with 98% prediction accuracy but suffers with computational complexity
**Crop quality** [14,46]	SVM using remotely sensed synthetic aperture radar (SAR) for paddy rice monitoring	Back-scattering features, SVM and regression tree with 77.65% accuracy; limited sample size
**Leaf area index** [55]	SAR images and machine learning and SVM	Gaussian process model, limited sample size
**Expert system for fertilizer, pesticides, irrigation control** [57]	Machine learning operates on sensor data	Naïve Bayes, 89.13% of accuracy; comparison of testing with different machine learning was missing
**Crop quality** [21,59,60]	Machine learning applied to real-time UAV images of soya bean crop. Tested 5 different diseases and soil quality assessment	Resnet-50, VGG-19 with 99.04 % accuracy
**Crop quality** [61]	Deep learning applied over Phenological data, 6 different crops were tested	CNN (convolutional neural network), accuracy not mentioned
**Smart farming** [62]	IoT, WSN, deep learning for fruit growth	SVM, accuracy not reported
**Pest control** [63]	IoT and deep learning using global and local features for pest monitoring	CNN model with 86.6% of average accuracy
**Crop area** [61]	Deep learning for plant area monitoring of peanut crop	CNN with 96.45% of accuracy

SVM: support vector machine; UAV: unmanned aerial vehicle

**Table 3:** Research on IoT based smart water pollution monitoring systems (SWPM).

Research	Purpose	Device/Method Used	Models
**Lagoon water** [64]	Agricultural water pollution control using remote sensing	Machine learning and image analysis for prediction	Linear regression (LR), stochastic gradient descent (SGD) and ridge regression (R-23 PLS)
**Water contamination** [65]	Water contamination assessments	FFT and machine learning	Color layout descriptor and SVM
**Water quality** [66]	Study of water pollutants	Extreme learning DSA-ELM model for classification	DSA-ELM model and dolphin swarm with 83.33% accuracy
**Water quality pollutants parameters** [67]	Water contamination analysis	Neural network for prediction for alkalinity, chloride, sulphate values	Levenberg–Marquardt algorithm with 87.23% accuracy
**Big data and SVM** [68]	Water contamination analysis	Machine learning based classification	SVM with 91.38% accuracy
**Drinking water** [69]	Drinking water analysis	Machine learning for classification: drinkable or non-drinkable water	DT, KNN, SVM with 97% accuracy
**Water quality** [70]	Water Contamination analysis	Neural network for classification: drinkable or non-drinkable water	SVM
**Water pollutant security** [71]	Water contamination surveillance	SVM for classification as polluted or clean water	SVM with 93.8% accuracy
**Drinking water** [73]	Drinking water analysis	Machine learning based prediction	FAST learning technique
**Chlorophyll-a in lake water** [72]	Chlorophyll-A concentration in lake water	machine learning based classification of water	BPNN, SVM with 78% accuracy
**Water quality monitoring** [74]	Water quality monitoring	IoT for surface water quality assessment	IoT with smart sensors

**Table 4:** Research on SAQM systems using machine learning and IoT.

Research	Purpose	Data and Technique
**Air quality characterization** [58]	Air quality monitoring	Heterogeneous sensors; machine learning based predictive model
**Air quality modeling** [75]	Air quality monitoring	Mobile nodes
**Air pollution** [76]	Air quality monitoring	Gas sensors from mobile vehicle data, IoT and machine learning
**Air quality in vehicular sensor network** [77]	Air quality monitoring	Sensors in mobile nodes
**Detection of VOC in air** [25]	Organic compound detection	Infrared sensors, spectroscopy and machine learning
**PM2.5 estimation** [78]	Air quality in terms of PM2.5 concentration levels	Spatio-temporal geographic data, Extreme machine learning technique
**Urban air** [44]	Urban air pollution in terms of O3, NO2 and SO2 concentrations	Forecasting models
**Air pollution prediction** [79]	Air pollution control	RFID, Gas sensors and IoT
**Smart air quality** [80]	Air quality	Temperature, humidity, dust and carbon dioxide sensor; LoRaWAN
**Intelligent air quality system** [83]	Air quality for detection of CO2, NOx, temperature and humidity	UV light, AI and sensors
**Ozone, PM10 and PM2.5** [84]	PM10, PM2.5, SO2, Oxides of nitrogen (NOx), O3, lead, CO and benzene	Machine learning and spatio-temporal data
**Air quality** [85]	Air quality	Heterogeneous sensors and SVM
**Abnormal O3** [84]	Ozone (O3)	Ozone data and deep learning
**Wearable sensors** [86]	Temperature and humidity monitoring	Wireless and wearable senor technology
**CO2 monitoring** [87]	Monitoring of carbon dioxide	IoT and cloud technologies
**Indoor air quality** [88]	Air quality monitoring in indoor environment	IoT, VOC: voloatile organic compound; LoRaWAN

(VOC: volatile organic compound; LoRaWAN: long range WAN)

**Table 5:** Quantum of research contributions using IoT and WSN; and IoT and machine learning.

Year	Research Using IoT and WSN	Research Using IoT and Machine Learning
1995–2000	21	2
2001–2005	7	7
2006–2010	22	2
2010–2015	541	175
2015–2020	6181	3004

## References

[1] Sayed E., Ahmed A., Yousef M.E. (2019). Internet of things in Smart Environment: Concept, Applications, Challenges, and Future Directions. World Sci. News.

[2] Jamil M.S., Jamil M.A., Mazhar A., Ikram A., Ahmed A., Munawar U. (2015). Smart Environment Monitoring System by Employing Wireless Sensor Networks on Vehicles for Pollution Free Smart Cities. Procedia Eng..

[3] Bhoomika K.N., Deepa C., Rashmi R.K.S. (2016). Internet of Things for Environmental Monitoring. Int. J. Adv. Netw. Appl..

[4] Cicala L., Angelino C.V., Parrilli S., Fiscante N., Liberata S., Addabbo P. (2018). Unsupervised Post-Fire Assessment of Burned Areas with Free and Open Multispectral Data Using OBIA. https://hal.univ-reunion.fr/hal-01957184.

[5] Gaglio S., Re G.L., Martorella G., Peri D., Vassallo S.D. Development of an IoT Environmental Monitoring Application with a Novel Middleware for Resource Constrained Devices. Proceedings of the 2nd Conference on Mobile and Information Technologies in Medicine (MobileMed 2014).

[6] Zhang D., Eng B., Prof S., Connor N.E.O., Regan P.F. (2015). Multi-Modal Smart Sensing Network for School of Electronic Engineering. Ph.D. Thesis.

[7] Tadejko P. (2017). Environmental monitoring systems using internet of things-standards and protocols Pawel Tadejko Environmental policy and management. Ekon. I Środowisko.

[8] Kulkarni P.H., Kute P.D. (2016). Internet of Things Based System for Remote Monitoring of Weather Parameters and Applications. Int. J. Adv. Electron. Comput. Sci..

[9] Kamal R. Lesson 11 Internet Connected Environment (Weather, Air Pollution and Forest Fire) Monitoring. https://www.dauniv.ac.in/public/frontassets/coursematerial/InternetofThings/IoTCh12L11EnvironmentMonitoring.pdf.

[10] Air Quality Monitoring Using IoT and Big Data GSMA 2018. https://www.gsma.com/iot/wp-content/uploads/2018/02/iot_clean_air_02_18.pdf.

[11] Jovanovska E.M., Davcev D. No pollution Smart City Sightseeing Based on WSN Monitoring System. Proceedings of the 2020 Sixth International Conference on Mobile And Secure Services (MobiSecServ).

[12] Arco E., Boccardo P., Gandino F., Lingua A., Noardo F., Rebaudengo M. (2016). An Integrated Approach for Pollution Monitoring: Smart Acquirement and Smart Information. ISPRS Ann. Photogramm. Remote Sens. Spat. Inf. Sci..

[13] Pavithra G. (2018). Journal of Agricultural Science and Intelligent Monitoring Device for Agricultural Greenhouse Using IOT. J. Agric. Sci. Food Res..

[14] Pathak A., Uddin M.A., Jainal Abedin M., Andersson K., Mustafa R., Hossain M.S. (2019). IoT based smart system to support agricultural parameters: A case study. Procedia Comput. Sci..

[15] Sivakannu G., Balaji S. (2017). Implementation of Smart Farm Monitoring Using IoT. Int. J. Curr. Eng. Sci. Res..

[16] Dhas Y.J., Jeyanthi P. (2017). Environmental Pollution Monitoring System Using Internet of Things (IoT). J. Chem. Pharm. Sci..

[17] Ullo S., Vaccaro A., Velotto G. The role of pervasive and cooperative sensor networks in smart grids communication. Proceedings of the 2010 15th IEEE Mediterranean Electrotechnical Conference (Melecon 2010).

[18] Morello R., De Capua C., Lugarà M. The design of a sensor network based on IoT technology for landslide hazard assessment. Proceedings of the 4th Imeko TC19 Symposium on Environmental Instrumentation and Measurements Protecting Environment, Climate Changes and Pollution Control.

[19] Gardner J. Smart Sensors in Mobile Phones for Environmental Monitoring. Proceedings of the Core-Group Meeting at Eurosensors—2014 Conference.

[20] Shahzadi R., Ferzund J., Tausif M., Asif M. (2016). Internet of Things based Expert System for Smart Agriculture. Int. J. Adv. Comput. Sci. Appl..

[21] Carminati M., Kanoun O., Ullo S.L., Marcuccio S. (2019). Prospects of Distributed Wireless Sensor Networks for Urban Environmental Monitoring. IEEE Aerosp. Electron. Syst. Mag..

[22] Kocakulak M., Butun I. An overview of Wireless Sensor Networks towards internet of things. Proceedings of the 2017 IEEE 7th Annual Computing and Communication Workshop and Conference (CCWC).

[23] LG Uplus Corp (2016). What Is IoT?.

[24] Gubbi J., Buyya R., Marusic S., Palaniswami M. (2013). Internet of Things (IoT): A vision, architectural elements, and future directions. Future Gener. Comput. Syst..

[25] Wong M.S., Wang T., Ho H.C., Kwok C.Y.T., Lu K., Abbas S. (2018). Towards a Smart City: Development and application of an improved integrated environmental monitoring system. Sustainability.

[26] Alharbi N., Soh B. (2019). Roles and Challenges of Network Sensors in Smart Cities. IOP Conf. Ser. Earth Environ. Sci..

[27] Nayyar A., Puri V. Smart farming: Iot based smart sensors agriculture stick for live temperature and moisture monitoring using arduino, cloud computing & solar technology. Proceedings of the International Conference on Communication and Computing Systems (ICCCS-2016).

[28] Kulkarni M.P.P. (2019). IOT based Smart Agricultural System. Int. J. Res. Appl. Sci. Eng. Technol..

[29] Shweta A.M., Nagaveni V. (2019). Survey on Smart Agriculture Using IOT. J. Comput. Program. Multimedia.

[30] Balakrishnan S., Vasudavan H., Murugesan R.K. Smart home technologies: A preliminary review. Proceedings of the 6th International Conference on Information Technology: IoT and Smart City (ICIT 2018).

[31] Mshali H., Lemlouma T., Moloney M., Magoni D. (2018). A survey on health monitoring systems for health smart homes. Int. J. Ind. Ergon..

[32] Duisebekova K.S., Tuyakova Z.N., Amanzholova S.T., Sarsenova Z.N., Duzbayev N.T., Pyagay V.T., Aitmagambetov A.Z. Environmental monitoring system for analysis of climatic and ecological changes using LoRa technology. Proceedings of the 5th International Conference on Engineering and MIS.

[33] Okafor N.U., Delaney D. (2019). Considerations for system design in IoT-based autonomous ecological sensing. Procedia Comput. Sci..

[34] Xu G., Shi Y., Sun X., Shen W. (2019). Internet of things in marine environment monitoring: A review. Sensors.

[35] Arora J., Pandya U., Shah S., Doshi N. (2019). Survey- Pollution monitoring using IoT. Procedia Comput. Sci..

[36] Li X., Liu Q., Yang R., Wen J., Zhang J., Cai E., Zhang H. (2016). The Combination of Ground-Sensing Network and Satellite Remote Sensing in Huailai County. IEEE Sens. J..

[37] Sharma J., John S. (2017). Real Time Ambient Air quality monitoring system using sensor technology. Int. J. Adv. Mech. Civ. Eng..

[38] Shelestov A., Kolotii A., Lavreniuk M., Medyanovskyi K., Bulanaya T., Gomilko I. Air Quality Monitoring in Urban Areas Using in-situ ind Satellite Data within Era-Planet Project Eos Data Analytics, Kyiv, Ukraine National Technical University of Ukraine “ Igor Sikorsky Kyiv Polytechnic Institute ”, Kyiv, Ukraine Space Research Insti. Proceedings of the International Geoscience and Remote Sensing Symposium (IGARSS 2018).

[39] Gallah N., Besbes K. Small satellite and multi-sensor network for real time control and analysis of lakes surface waters. Proceedings of the RAST 2013: 6th Conference on Recent Advances in Space Technologies.

[40] Shaikh S.F., Hussain M.M. Marine IoT: Non-invasive wearable multisensory platform for oceanic environment monitoring. Proceedings of the IEEE 5th World Forum Internet Things (WF-IoT 2019).

[41] Daniels E.T., McPheron B.D. A machine learning approach to classifying algae concentrations. Proceedings of the 2017 IEEE MIT Undergraduate Research Technology Conference (MA URTC 2017).

[42] Durante G., Beccaro W., Peres H.E.M. (2018). IoT Protocols Comparison for Wireless Sensors Network Applied to Marine Environment Acoustic Monitoring. IEEE Lat. Am. Trans..

[43] Al Mamun M.A., Yuce M.R. (2019). Sensors and Systems for Wearable Environmental Monitoring Toward IoT-Enabled Applications: A Review. IEEE Sens. J..

[44] Shaban K.B., Kadri A., Rezk E. (2016). Urban air pollution monitoring system with forecasting models. IEEE Sens. J..

[45] Goodson L.H., Jacobs W.B., Davis A.W. (1974). Air Pollution Monitoring System. Pesticides Abstracts.

[46] Shinde D., Siddiqui N. IOT Based Environment change Monitoring Controlling in Greenhouse using WSN. Proceedings of the 2018 International Conference on Information, Communication, Engineering and Technology (ICICET 2018).

[47] Dhingra S., Madda R.B., Gandomi A.H., Patan R., Daneshmand M. (2019). Internet of things mobile-air pollution monitoring system (IoT-Mobair). IEEE Internet Things J..

[48] Beebi F. (2018). Environmental Monitoring System Using IoT. India Res. Tech. Organiz..

[49] Chen W.P., Wang L.K., Wang T.T., Chen Y.T. (2013). An Intelligent Management System for Aquacultures Environmental Monitoring and Energy Conservation. Advances in Intelligent Systems Research.

[50] Mocanu I., Florea A.M. A multi-agent supervising system for smart environments. Proceedings of the 2nd International Conference on Web Intelligence, Mining and Semantics.

[51] Yamashita K., Ao C., Suzuki T., Xu Y., Li H., Tian J., Kimura K., Kasahara H. Architecture design for the environmental monitoring system over the winter season. Proceedings of the 14th ACM International Symposium on Mobility Management and Wireless Access (MobiWac 2016).

[52] Santos D., Mataloto B., Ferreira J.C. (2019). Data center environment monitoring system. ACM Int. Conf. Proc. Ser..

[53] Chehri A., Saadane R. (2019). Zigbee-based remote environmental monitoring for smart industrial mining. ACM Int. Conf. Proc. Ser..

[54] Kumar S., Chowdhary G., Udutalapally V., Das D., Mohanty S.P. GCrop: Internet-of-Leaf-Things (IoLT) for monitoring of the growth of crops in smart agriculture. Proceedings of the 5th IEEE International Symposium on Smart Electronic Systems (Formerly iNIS) (IEEE-iSES 2019).

[55] Hosseini M., McNairn H., Mitchell S., Davidson A., Robertson L.D. Comparison of Machine Learning Algorithms and Water Cloud Model for Leaf Area Index Estimation Over Corn Fields. Proceedings of the IGARSS 2019 - 2019 IEEE Int. Geosci. Remote Sens. Symp.

[56] Fazai R., Mansouri M., Abodayeh K., Puig V., Selmi M., Nounou H., Nounou M. (2019). Multiscale Gaussian Process Regression-Based GLRT for Water Quality Monitoring. Conf. Control Fault Toler. Syst. Sys. Tol..

[57] Dimitriadis S., Goumopoulos C. Applying machine learning to extract new knowledge in precision agriculture applications. Proceedings of the 12th Pan-Hellenic Conference on Informatics Doryssa Seaside Resort (PCI 2008).

[58] Amado T.M., Cruz J.C. Dela Development of Machine Learning-based Predictive Models for Air Quality Monitoring and Characterization. Proceedings of the TENCON 2018, 2018 IEEE Reg.

[59] Saha A.K., Saha J., Ray R., Sircar S., Dutta S., Chattopadhyay S.P., Saha H.N. IOT-based drone for improvement of crop quality in agricultural field. Proceedings of the 8th IEEE Annual Computing and Communication Workshop and Conference (IEEE CCWC).

[60] Di Martini D.R., Liesenberg V., Tetila E.C., Junior J.M., Matsubara E.T., Siqueira H., De Castro Junior A.A., Araujo M.S., Monteiro C.H., Pistori H. Machine Learning Applied to UAV Imagery in Precision Agriculture and Forest Monitoring in Brazililian Savanah. Proceedings of the International Geoscience and Remote Sensing Symposium 2019 (IGARSS 2019).

[61] Zhou Z., Li S. Peanut planting area change monitoring from remote sensing images based on deep learning. Proceedings of the 2017 4th International Conference on Systems and Informatics (ICSAI 2017).

[62] Boursianis A.D., Papadopoulou M.S., Diamantoulakis P., Liopa-Tsakalidi A., Barouchas P., Salahas G., Karagiannidis G., Wan S., Goudos S.K. (2020). Internet of Things (IoT) and Agricultural Unmanned Aerial Vehicles (UAVs) in Smart Farming: A Comprehensive Review. Internet Things.

[63] Liu L., Wang R., Xie C., Yang P., Sudirman S., Wang F., Li R. (2019). Deep learning based automatic approach using hybrid global and local activated features towards large-scale multi-class pest monitoring. IEEE Int. Conf. Ind. Inform..

[64] Li Y., Wang X., Zhao Z., Han S., Liu Z. (2020). Lagoon water quality monitoring based on digital image analysis and machine learning estimators. Water Res..

[65] Chen Q., Cheng G., Fang Y., Liu Y., Zhang Z., Gao Y., Horn B.K.P. Real-time Learning-based Monitoring System for Water Contamination. Proceedings of the 2018 4th International Conference on Universal Village (UV 2018).

[66] Yan H., Liu Y., Han X., Shi Y. An evaluation model of water quality based on DSA-ELM method. Proceedings of the 16th International Conference on Optical Communications and Networks (ICOCN 2017).

[67] Ragi N.M., Holla R., Manju G. Predicting Water Quality Parameters Using Machine Learning. Proceedings of the 4th IEEE International Conference on Recent Trends on Electronics, Information & Communication Technology (RTEICT-2019).

[68] Budiarti R.P.N., Sukaridhoto S., Hariadi M., Purnomo M.H. Big Data Technologies using SVM (Case Study: Surface Water Classification on Regional Water Utility Company in Surabaya). Proceedings of the 2019 International Conference on Computer Science, Information Technology, and Electrical Engineering (ICOMITEE 2019).

[69] Jalal D., Ezzedine T. Toward a smart real time monitoring system for drinking water based on machine learning. Proceedings of the The 27 th International Conference on Software, Telecommunications and Computer Networks (SoftCOM 2019).

[70] Bouamar M., Ladjal M. (2007). Evaluation of the performances of ANN and SVM techniques used in water quality classification. Proc. IEEE Int. Conf. Electron. Circuits Syst..

[71] Pang Z., Jia K., Feng J. A water environment security monitoring algorithm based on intelligent video surveillance. Proceedings of the 2014 Tenth International Conference on Intelligent Information Hiding and Multimedia Signal Processing (IIH-MSP 2014).

[72] Liu J., Zhang Y., Qian X. Modeling chlorophyll-a in Taihu Lake with machine learning models. Proceedings of the The 3rd International Conference on Bioinformatics and Biomedical Engineering (iCBBE 2009).

[73] Imen S., Chang N.B., Yang Y.J., Golchubian A. (2018). Developing a Model-Based Drinking Water Decision Support System Featuring Remote Sensing and Fast Learning Techniques. IEEE Syst. J..

[74] Asiful Islam M., Khan R.H., Syeed M. (2020). A smart and integrated surface water monitor system architecture: Bangladesh perspective. ACM Int. Conf. Proc. Ser..

[75] Mihăiţă A.S., Dupont L., Chery O., Camargo M., Cai C. (2019). Evaluating air quality by combining stationary, smart mobile pollution monitoring and data-driven modelling. J. Clean. Prod..

[76] Shetty C., Sowmya B.J., Seema S., Srinivasa K.G. (2020). Air Pollution Control Model Using Machine Learning and IoT Techniques.

[77] Van Le D., Tham C.K. Machine learning (Ml)-based air quality monitoring using vehicular sensor networks. Proceedings of the 38th IEEE International Conference on Distributed Computing Systems.

[78] Liu B., Yan S., Li J., Li Y. Forecasting PM2.5 Concentration Using Spatio-Temporal Extreme Learning Machine. Proceedings of the 15th IEEE International Conference on Machine Learning and Applications (IEEE ICMLA’16).

[79] Ayele T.W., Mehta R. Air pollution monitoring and prediction using IoT. Proceedings of the 2nd International Conference on Inventive Communication and Computational Technologies (ICICCT 2018).

[80] Thu M.Y., Htun W., Aung Y.L., Shwe P.E.E., Tun N.M. Smart air quality monitoring system with LoRaWAN. Proceedings of the 2018 International Conference on Internet of Things and Intelligence System (IoTaIS 2018).

[81] Ou C.H., Chen Y.A., Huang T.W., Huang N.F. (2020). Design and Implementation of Anomaly Condition Detection in Agricultural IoT Platform System. Int. Conf. Inf. Netw..

[82] Deng F., Zuo P., Wen K., Wu X. (2020). Novel soil environment monitoring system based on RFID sensor and LoRa. Comput. Electron. Agric..

[83] Rosero-Montalvo P.D., Caraguay-Procel J.A., Jaramillo E.D., Michilena-Calderon J.M., Umaquinga-Criollo A.C., Mediavilla-Valverde M., Ruiz M.A., Beltran L.A., Peluffo-Ordónez D.H. Air quality monitoring intelligent system using machine learning techniques. Proceedings of the 3rd International Conference on Information, Systems and Computer Science (INCISCOS 2018).

[84] Chiwewe T.M., Ditsela J. (2016). Machine learning based estimation of Ozone using spatio-temporal data from air quality monitoring stations. IEEE Int. Conf. Ind. Informatics.

[85] Ali S., Tirumala S.S., Sarrafzadeh A. SVM aggregation modelling for spatio-temporal air pollution analysis. Proceedings of the ACM MobiSys 2015 Workshop on Wearable Systems and Applications.

[86] Cho H. Design and implementation of a wearable environmental monitoring system. Proceedings of the ACM MobiSys 2015 Workshop on Wearable Systems and Applications.

[87] Ming F.X., Ariyaluran Habeeb R.A., Md Nasaruddin F.H.B., Gani A. (2019). Bin Real-time carbon dioxide monitoring based on IoT & cloud technologies. ACM Int. Conf. Proc. Ser..

[88] AbdulWahhab R.S. (2019). Air quality system using IoT for indoor environmental monitoring. ACM Int. Conf. Proc. Ser..

[89] Ameer S., Shah M.A., Khan A., Song H., Maple C., Islam S.U., Asghar M.N. (2019). Comparative Analysis of Machine Learning Techniques for Predicting Air Quality in Smart Cities. IEEE Access.

[90] Srikamdee S., Onpans J. Forecasting Daily Air Quality in Northern Thailand Using Machine Learning Techniques. Proceedings of the 4th International Conference on Information Technology (InCIT2019).

[91] Ghanshala K.K., Chauhan R., Joshi R.C. A Novel Framework for Smart Crop Monitoring Using Internet of Things (IOT). Proceedings of the First International Conference on Secure Cyber Computing And Communications (ICSCCC 2018).

[92] Gartia M.R., Braunschweig B., Chang T.W., Moinzadeh P., Minsker B.S., Agha G., Wieckowski A., Keefer L.L., Liu G.L. (2012). The microelectronic wireless nitrate sensor network for environmental water monitoring. J. Environ. Monit..

[93] Nascimento Silva H.A., Panella M. (2018). Eutrophication Analysis of Water Reservoirs by Remote Sensing and Neural Networks. Prog. Electromagn. Res. Symp..

[94] Marino R., Quintero S., Lanza-gutierrez J.M., Riesgo T., Holgado M., Portilla J., Torre E. De Water Media based on Machine Learning Techniques. Proceedings of the 2019 XXXIV Conference on Design of Circuits and Integrated Systems (DCIS).

[95] Shafi U., Mumtaz R., Anwar H., Qamar A.M., Khurshid H. Surface Water Pollution Detection using Internet of Things. Proceedings of the 2018 International Conference on High-capacity Optical Networks & Enabling/Emerging Technologies (HONET-ICT 2018).

[96] Dang C.L., Yang J., Zhang X.Y., Li S.F. The application of the fuzzy attenuation model in the evaluation of water quality in the Yangtze River. Proceedings of the ICMLC 2008: International Conference on Machine Learning and Cybernetics (ICMLC 2008).

[97] Addabbo P., Focareta M., Marcuccio S., Votto C., Ullo S.L. (2016). Contribution of Sentinel-2 data for applications in vegetation monitoring. Acta IMEKO.

[98] Mazǎre A.G., Lonescu L.M., Liţa I., Vişan D., Belu N., Gherghe M. Intelligent monitoring and planning system for herbicidal processes in agricultural crops. Proceedings of the 2018 IEEE 24th International Symposium for Design and Technology in Electronic Packaging (SIITME 2018).

[99] Kucuk C., Kaya G.T., Erten E. (2015). CO-POLAR SAR data classification as a tool for real time paddy-rice monitoring. Int. Geosci. Remote Sens. Symp..

[100] Agarwal A., Kumar S., Singh D. (2019). Development of Machine Learning Based Approach for Computing Optimal Vegetation Index with the Use of Sentinel-2 and Drone Data. Int. Geosci. Remote Sens. Symp..

[101] Sharma H., Haque A., Jaffery Z.A. (2019). Maximization of wireless sensor network lifetime using solar energy harvesting for smart agriculture monitoring. Ad Hoc Netw..

[102] Kanaan M., Bavkara C.K. Proactive Monitoring and Classification of Stored Grain Condition via Wireless Sensor Networks and Machine Learning Techniques. Proceedings of the 2018 2nd International Symposium on Multidisciplinary Studies and Innovative Technologies (ISMSIT 2018).

[103] Hossain M.A., Atrey P.K., El Saddik A. (2011). Modeling and assessing quality of information in multisensor multimedia monitoring systems. ACM Trans. Multimed. Comput. Commun. Appl..

[104] Mukherji S.V., Sinha R., Basak S., Kar S.P. Smart Agriculture using Internet of Things and MQTT Protocol. Proceedings of the 2019 International Conference on Machine Learning, Big Data, Cloud and Parallel Computing (COMITCon).

[105] Mois G., Folea S., Sanislav T. (2017). Analysis of Three IoT-Based Wireless Sensors for Environmental Monitoring. IEEE Trans. Instrum. Meas..

[106] Glaroudis D., Iossifides A., Chatzimisios P. (2020). Survey, comparison and research challenges of IoT application protocols for smart farming. Comput. Netw..

[107] Alsamhi S.H., Ma O., Ansari M.S., Meng Q. (2018). Greening Internet of Things for Smart Everythings with a Green-Environment Life: A Survey and Future Prospects. Signal Process..

[108] Marcuccio S., Ullo S., Carminati M., Kanoun O. (2019). Smaller Satellites, Larger Constellations: Trends and Design Issues for Earth Observation Systems. IEEE Aerosp. Electron. Syst. Mag..

[109] Ullo S., Gallo M., Palmieri G., Amenta P., Russo M., Romano G., Ferrucci M., Ferrara A., De Angelis M. Application of wireless sensor networks to environmental monitoring for sustainable mobility. Proceedings of the 2018 IEEE International Conference on Environmental Engineering (EE).

[110] Ullo S.L., Addabbo P., Di Martire D., Sica S., Fiscante N., Cicala L., Angelino C.V. (2019). Application of DInSAR Technique to High Coherence Sentinel-1 Images for Dam Monitoring and Result Validation Through in Situ Measurements. IEEE J. Sel. Top. Appl. Earth Obs. Remote Sens..

[111] Ullo S.L., Langenkamp M.S., Oikarinen T.P., Delrosso M.P., Sebastianelli A., Iccirillo F.P., Sica S. (2019). Landslide Geohazard Assessment with Convolutional Neural Networks Using Sentinel-2 Imagery Data. Int. Geosci. Remote Sens. Symp..

[112] Cicala L., Angelino C.V., Fiscante N., Ullo S.L. (2018). Landsat-8 and Sentinel-2 for fire monitoring at a local scale: A case study on Vesuvius. IEEE Int. Conf. Environ. Eng..

[113] Addabbo P., Focareta M., Marcuccio S., Votto C., Ullo S.L. (2016). Land cover classification and monitoring through multisensor image and data combination. Int. Geosci. Remote Sens. Symp..

